# Increased risk of subsequent benign prostatic hyperplasia in non-Helicobacter pylori-infected peptic ulcer patients: a population-based cohort study

**DOI:** 10.1038/s41598-020-78913-y

**Published:** 2020-12-10

**Authors:** Chu-Wen Fang, Chun-Hao Chen, Kun-Hung Shen, Wen-Chi Yang, Chih-Hsin Muo, Shih-Chi Wu

**Affiliations:** 1 Division of Urology, Department of Surgery, Chi Mei Medical Center, Tainan, Taiwan; 2 Division of Hematology and Medical Oncology, Department of Internal Medicine, E-DA Hospital, Kaohsiung, Taiwan; 3 Faculty of School of Medicine, College of Medicine, I-Shou University, Kaohsiung, Taiwan; 4 Management Office for Health Data, China Medical University Hospital, Taichung, Taiwan; 5 School of Medicine, China Medical University, Taichung, Taiwan; 6 Trauma and Emergency Center, China Medical University Hospital, No. 2, Yuh-Der Road, Taichung, 404 Taiwan

**Keywords:** Diseases, Health care

## Abstract

The vagus nerve plays an essential role in homeostasis and inflammation. Clinically, peptic ulcer patients without helicobacter pylori (HP) infection may provide a population for studying the effect of vagal hyperactivity. There were interests in the association of gastrointestinal disease and urogenital disorders. Herein, we try to investigate subsequent risk of benign prostatic hyperplasia (BPH) in non-HP infected peptic ulcer patients. We identified 17,672 peptic ulcer admission male patients newly diagnosed in 1998–2007 from Taiwan Health Insurance Database, and 17,672 male comparison without peptic ulcer, frequency matched by age, and index-year. We assessed subsequent incidence of BPH in each cohort by the end of 2013, and then compared the risk of developing BPH between individuals with and without peptic ulcer. In addition, peptic ulcer patients underwent surgery were also examined. There were 2954 peptic ulcer patients and 2291 comparisons noted with the occurrence of BPH (25.35 and 16.70 per 1000 person-years, respectively). Compared to comparisons, peptic ulcer patients had a 1.45- and 1.26-fold BPH risk in multivariable Cox model and Fine and Gray model (95% CI 1.37–1.54 and 1.19–1.34). In age-stratified analysis, the highest risk of BPH was in 45–59 years (interaction p < 0.05). Regarding surgery types, peptic ulcer patients who underwent simple suture surgery (i.e.: with integrated vagus nerve) had a significant higher BPH risk than comparison (HR 1.50 and 95% CI 1.33–1.74; SHR 1.26 and 95% CI 1.07–1.48), while patients underwent truncal vagotomy/pyloroplasty showed a lower incidence of BPH. In this study, non-HP-infected male peptic ulcer patients were found to have an increased risk of subsequent BPH. Indicating that there might be a role of vagus nerve. Based on the limitations of retrospective nature, further studies are required.

## Introduction

Peptic ulcer disease is mucosal damage of the gastrointestinal tract owing to pepsin and gastric acid secretion^1^. It was a prevailing disease in the world and the incidence of uncomplicated peptic ulcer is about one case per 1000 person-years in the general population, while the incidence of ulcer complications was about 0.7 cases per 1000 person-years^2^. In addition, there were reported about 500,000 newly developed peptic ulcer disease in the United States each year, the direct and indirect costs of the disease are estimated at about $10 billion each year^1,3^.

The vagus nerve plays an essential role in maintaining metabolic homeostasis and regulation of inflammation^4,5^. Human studies showed that an increased vagal parasympathetic tone was associated with peptic ulcer formation^6,7^, while vagal hyperactivity was related to gastric stress ulceration development in rodent study^8^. Moreover, cohort studies have reported that increased risks of inflammatory diseases are noted in peptic ulcer patients, such as type 2 diabetes^9^, ischemic heart disease^10^, ischemic stroke^11,12^, and liver cirrhosis^13^. Indicating that persistent vagal hyperactivity might play a role in these inflammatory diseases^9,10^.

On the other hand, benign prostatic hyperplasia (BPH) is a common urological disorder in males that involve unregulated proliferation of glandular epithelium and connective tissue within the prostatic transition zone^14^, which may cause gradual obstruction of the bladder outflow and result in urgency, frequency, nocturia and other obstructive symptoms that are well recognized as lower urinary tract symptoms (LUTS)^15^.

There is an interest in the association between gastrointestinal diseases and urological diseases, studies focused on the impact of helicobacter pylori (HP) infection and urological diseases^16–18^. However, few studies addressed on the association of persistent vagal hyperactivity and the development of urological diseases. In addition, peptic ulcer patients without helicobacter pylori (HP) infection may provide a population group for studying the effect of vagal hyperactivity. Therefore, we were interested in this issue and try to investigate subsequent risk of BPH in non-HP infected peptic ulcer patients.

## Data source

We used a Longitudinal Health Insurance Database (LHID) from Taiwan National Health Insurance (TNHI) program that was set up by Ministry of Health and Welfare. There are over 99% of population in Taiwan joining this TNHI program. The details of the LHID have been reported in previous studies^19–21^. The LHID contained all medical records between January 1, 1996 and December 31, 2013 for one million insurant that were randomly selected from TNHI program. As a result, we collected patients and classify the disease and treatment in LHID based on the International Classification of Diseases, Ninth Revision, Clinical Modification (ICD-9-CM).

### Study subject

We collected patients with newly peptic ulcer hospitalization (ICD-9-CM 531–533) from 1998 to 2007 as peptic ulcer cohort in this retrospective cohort study. The index date was defined as the date for peptic ulcer hospitalization. The exclusion criteria include: 1. Age < 18 years; 2. with documented HP infection; 3. with BPH history. To reduce the effect of causal inversion, we also excluded patients with BPH development within one year.

**Figure 1:**
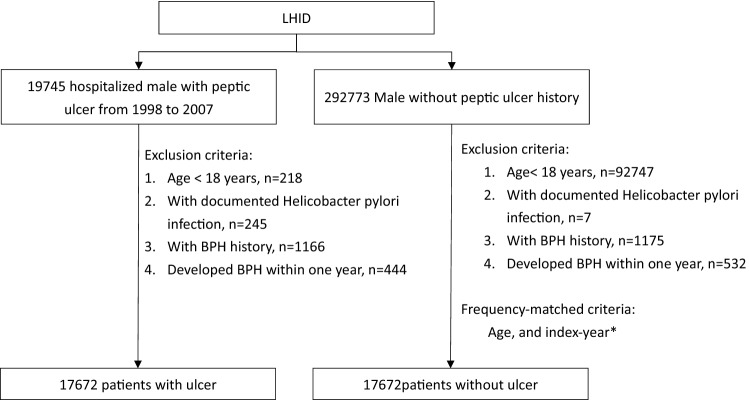
Flow chart for selecting study cohorts. *Randomly assigned entry date as ulcer admission date.

In addition, the comparison cohort was selected from males without a history of peptic ulcer in LHID. The exclusion criteria for the comparison cohort were that same as those for peptic ulcer cohort. The comparisons were frequency matched with peptic ulcer patients at a 1:1 ratio based on age, and index-year. The details are presented in Fig. 1.

To comply with the Personal Information Protection Act, all of the identifying information of the insured people were removed and replaced with surrogate numbers for research use. All study methods were performed in accordance with the relevant guidelines and regulations. In addition, the need of informed consent was waived off by the Ethics Committee. This study was approved by the Research Ethics Committee of China Medical University and Hospital in Taiwan [CMUH104-REC2-115(CR-1)].

### Outcome, baseline comorbidity and surgery

The anticipated outcome was subsequent BPH development (ICD-9-CM 600.0).

The diagnosis of BPH was confirmed by specialists based on the guideline from the Taiwan National Health Insurance Administration Ministry of Health and Welfare.

In addition, all study subjects were followed from the index date until the date of diagnosis of BPH, withdrew from the LHID, or the end of 2013, whichever came first. Furthermore, the considered comorbidity in this study include diabetes mellitus (DM), asthma, obesity diagnosis (BMI > 27 kg/m^2^) (ICD-9-CM 278.0), chronic obstructive pulmonary disease (COPD), coronary artery disease (CAD), stroke, and alcohol-related disease (ALD, which include cirrhosis, alcoholic psychoses, alcohol dependence syndrome, alcohol abuse, alcoholic fatty live, acute alcoholic hepatitis, alcoholic cirrhosis, and alcoholic liver damage). In peptic ulcer patients underwent surgery, the peptic ulcer-associated surgeries were grouped into simple suture, truncal vagotomy/pyloroplasty (TVP), and others.

### Statistical analysis

The Chi-square test was used to test the differences of distribution for age group (18–44, 45–59, 60–74, and 75 + years), and comorbidity between peptic ulcer and comparison cohort. T-test was used to test the different of mean age between two cohorts. The BPH development rate in two cohorts was respectively calculated: the sum of BPH development was divided by the sum of follow-up duration (person-years). The hazard ratio (HR) and 95% confidence interval (CI) was assessed by Cox proportional hazard regression in peptic ulcer cohort when compared with comparison cohort. In addition, the multivariable Cox model was adjusted for age, DM, asthma, COPD, CAD, stroke, and ALD.

Because the mortality in peptic ulcer cohort was higher than those in comparison cohort (14.4% vs. 7.12%), the sub-distribution hazard ratio (SHR) was also estimated by competing risk regression based on Fine and Gray method. We also assessed the BPH risk stratified by age group, and all comorbidities. Because the study was violating the assumption of Cox proportional hazard regression based on the Schoenfeld residuals, the association between BPH and peptic ulcer was estimated stratified by follow-up years.

Finally, we assessed the association between BPH and different peptic ulcer-associated surgery. The Kaplan–Meier analysis was used to plot the cumulative incidence for BPH development and the log-rank test was used to test the difference of cumulative incidence. All statistical analyses were used on SAS software version 9.4 (SAS Institute, Cary, NC). The significant level was set at p < 0.05 under two-tailed tests.

## Result

We collected 17,672 hospitalized males with peptic ulcer and 292,773 male comparisons without peptic ulcer history from LHID. After exclusion and frequency matched, a total of 17,672 peptic ulcer male patients and 17,672 male comparisons were enrolled in this study. The mean age in peptic ulcer patients was 57.8 years (standard deviation = 17.6) (Table 1). Compared to comparisons, peptic ulcer patients were likely with higher prevalence of DM (21.6% vs. 10.9%), COPD (39.1% vs. 20.7%), asthma (9.21% vs. 3.36%), CAD (24.5% vs. 10.8%), stroke (22.2% vs. 10.2%), and ALD (12.7% vs. 1.71%).

**Table 1:** Demographic status, and comorbidity of study cohorts.

Variable	Peptic ulcer N = 17,672		Comparison N = 17,672		p-value
	N	%	n	%	
**Age, year**					0.99
18–44	4567	25.8	4567	25.8	
45–59	4334	24.5	4334	24.5	
60–74	5465	30.9	5465	30.9	
75 +	3306	18.7	3306	18.7	
Mean (SD)	57.8	(17.6)	57.7	(17.6)	0.57
**Comorbidity**					
DM	3819	21.6	1917	10.9	< 0.0001
COPD	6915	39.1	3651	20.7	< 0.0001
Asthma	1627	9.21	593	3.36	< 0.0001
CAD	4324	24.5	1910	10.8	< 0.0001
Stroke	3738	21.2	1793	10.2	< 0.0001
ALD	2238	12.7	303	1.71	< 0.0001

*ALD* alcohol-related disease, *CAD* coronary artery disease, *COPD* chronic obstructive pulmonary disease, *DM* diabetes mellitus, *SD* standard deviation.

Chi-square test, and †t-test.

**Figure 2:**
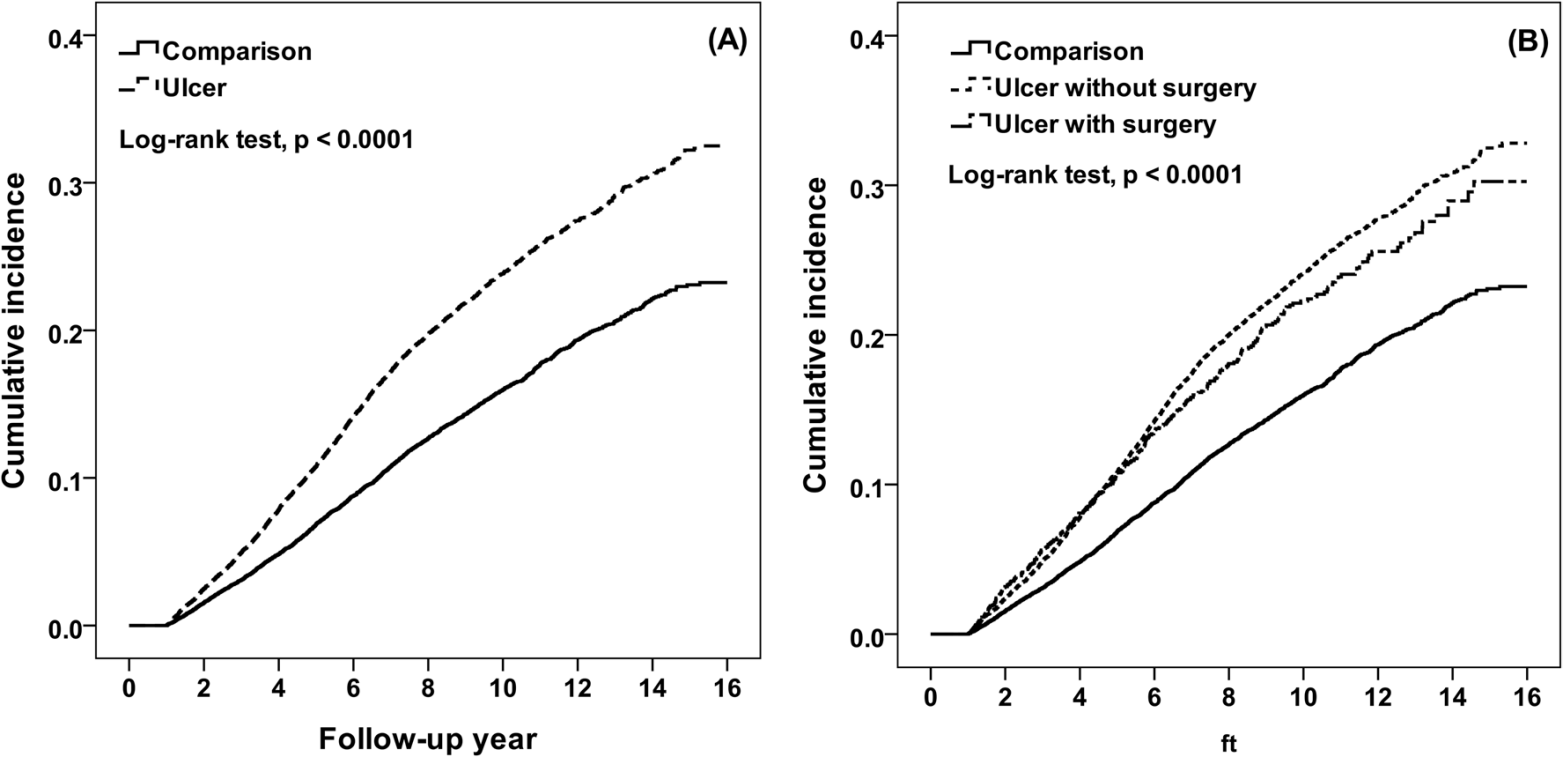
Kaplan–Meier analysis measured cumulative incidence of benign prostatic hyperplasia for comparison cohort and ulcer cohort.

During the study period, there were 2954 and 2291 BPH patients identified in the peptic ulcer and comparison cohort, which corresponds to 25.35 and 16.70 per 1000 person-years (Table 2). The cumulative BPH incidence in peptic ulcer cohort was 9.26% higher than that in the comparison cohort after a follow-up of 16 years (32.50% vs. 23.24%, respectively. Figure 2A). Compared to the comparison cohort, peptic ulcer patients had a 1.45- and 1.26-fold BPH risk in multivariable Cox model and Fine and Gray model (95% CI 1.37–1.54 and 1.19–1.34), respectively, (Table 2).

**Table 2:** Incidence and Cox proportional hazards regression method estimated hazard ratio of BPH by sex, age, comorbidity and follow-up.

Variable	Peptic ulcer		Comparison		HR (95% CI)^a^	SHR (95% CI)^b^
	Case	Rate	Case	Rate		
Overall	2954	25.35	2291	16.70	1.45 (1.37–1.54)***	1.26 (1.19–1.34)***
**Age, year**^**#**^						
18–44	140	3.15	75	1.69	1.44 (1.07–1.95)*	1.39 (1.02–1.90)**
45–59	715	22.13	440	10.92	1.73 (1.52–1.97)***	1.63 (1.43–1.85)***
60–74	1436	49.44	1199	31.67	1.34 (1.23–1.46)***	1.23 (1.13–1.33)***
75 +	663	61.61	577	39.61	1.34 (1.19–1.51)***	1.12 (0.99–1.27)
**DM**^**#**^						
No	2169	22.44	1923	15.23	1.47 (1.38–1.58)***	1.26 (1.18–1.35)***
Yes	785	39.55	368	33.74	1.31 (1.15–1.49)***	1.16 (1.02–1.32)*
**COPD**						
No	1343	16.93	1566	13.65	1.51 (1.40–1.63)***	1.31 (1.21–1.41)***
Yes	1611	43.31	725	32.26	1.38 (1.26–1.51)***	1.21 (1.11–1.33)***
**Asthma**						
No	2547	23.45	2188	16.34	1.43 (1.34–1.52)***	1.24 (1.17–1.32)***
Yes	407	51.55	103	30.91	1.80 (1.44–2.25)***	1.54 (1.24–1.91)***
**CAD**						
No	1787	18.90	1857	14.61	1.48 (1.38–1.58)***	1.25 (1.16–1.34)***
Yes	1167	53.06	434	42.78	1.33 (1.18–1.49)***	1.22 (1.09–1.36)***
**Stroke**^**#**^						
No	2123	21.27	1941	15.04	1.49 (1.40–1.59)***	1.29 (1.21–1.38)***
Yes	831	49.70	350	42.75	1.23 (1.08–1.40)**	1.08 (0.96–1.23)
**ALD**						
No	2725	26.47	2264	16.73	1.45 (1.37–1.54)***	1.26 (1.19–1.34)***
Yes	229	16.85	27	14.28	1.80 (1.20–2.72)**	1.56 (1.03–2.36)*
**Follow-up years**						
< 3	674	15.92	461	9.99	1.32 (1.16–1.50)***	1.21 (1.07–1.38)***
4–5	715	31.70	520	19.79	1.39 (1.23–1.57)***	1.37 (1.21–1.55)***
> 5	1565	30.31	1310	20.22	1.55 (1.43–1.68)***	1.51 (1.39–1.63)***

Rate, per 1000 person-years.

*ALD* alcohol-related disease, *BPH* benign prostatic hyperplasia, *CAD* coronary artery disease, *CI* confidence interval, *COPD* chronic obstructive pulmonary disease, *DM* diabetes mellitus, *HR* hazard ratio, *SHR* subdistribution hazard ratio.

^#^Interaction p < 0.05 in model 1. Cox proportiona assumption test, p = 0.003.

*p < 0.05, **p < 0.01, ***p < 0.001.

^a^Using Cox proportional hazard regression after adjustingd for age, and all comorbidity.

^b^Using Cox proportional hazard regression with competing risk (death) after adjusting for age, and all comorbidity.

In age-stratified analysis, the highest risk of BPH was in 45–59 years (HR 1.73 and 1.63, 95% CI 1.52–1.97 and 1.43–1.85 in multivariable Cox model and Fine and Gray model, respectively), and follow-up by 18–44 years, 60–74 years, and 75 + years (Table 2). In comorbidity-stratified analysis, study subjects without comorbidities had a higher BPH risk than those with comorbidities except for asthma and ALD. However, only DM and stroke showed statistical significances (interaction test p < 0.05). In follow-up duration-stratified analysis, BPH risk was noted the highest in > 5 years, and the lowest in < 3 years.

Table 3 showed the association between BPH and peptic ulcer patients underwent surgery. Peptic ulcer patients without surgery showed the highest BPH incidence (25.58 per 1000 person-years), followed by peptic ulcer patients with surgery (23.70 per 1000 person-years), and comparisons (16.70 per 1000 person-years). At the follow-up 16 years, the cumulative incidence of BPH in peptic ulcer patients without surgery and with surgery were higher than comparisons (23.24% and 32.81% vs. 30.25%) (Fig. 2B). When compared to the comparison cohort, the peptic patients without surgery had a higher BPH risk (HR 1.46 and 95% CI 1.38–1.55; SHR 1.26 and 95% CI 1.19–1.34), followed by those with surgery (HR 1.39 and 95% CI 1.24–1.57; SHR 1.24 and 95% CI 1.10–1.40) (Table 3).

**Table 3:** Multivariable Cox method measured hazard ratio of BPH in comparison cohort and peptic ulcer patients by surgery.

Surgery	N	Case	Rate	HR (95% CI)^a^	SHR (95% CI)^b^
Comparison	17,672	2291	16.70	1.00	1.00
**Peptic ulcer management**					
Without surgery	15,521	2615	25.58	1.46 (1.38–1.55)***	1.26 (1.19–1.34)***
With surgery	2151	339	23.70	1.39 (1.24–1.57)***	1.24 (1.10–1.40)***
TVP	360	41	12.56	1.04 (0.76–1.42)	0.95 (0.70–1.81)
Simple suture	1699	285	27.47	1.50 (1.33–1.70)***	1.33 (1.17–1.51)***
Other	92	13	19.61	0.95 (0.55–1.63)	0.91 (0.52–1.59)

*HR* hazard ratio, *CI* confidence interval, *SHR* subdistribution hazard ratio, *TVP* truncal vagotomy and pyloroplasty.

Rate, per 1000 person-years.

*p < 0.05, **p < 0.01, ***p < 0.001.

^a^Using Cox proportional hazard regression after adjusting for age, and all comorbidity.

^b^Using Cox proportional hazard regression with competing risk (death) after adjusting for age, and all comorbidity.

Among different peptic ulcer associated-surgery, patients with simple suture had a significant higher BPH risk when compared to comparisons (HR 1.50 and 95% CI 1.33–1.70; SHR 1.33 and 95% CI 1.17–1.51). However, patients underwent TVP and other surgery were with a lower incidence of BPH, but without significance.

## Discussion

BPH is a common urological disorder in males that involve unregulated proliferation of glandular epithelium and connective tissue within the prostatic transition zone^14^, while the potentially risk factors for BPH may include diabetes, obesity and lower urinary tract symptoms^22^. The pathophysiology of BPH may include androgenic stimulation resulted prostate gland overgrowth, and increased adrenergic tone leading to smooth muscle contraction^23,24^. Thus, medications for BPH may include anti-androgenic drugs and anti-adrenergic drugs^25,26^.

Additionally, vagal hyperactivity was reported to be associated with the development of peptic ulcer disease^8,27^. In this large cohort study, the peptic ulcer patients provided an appropriate population group for studying the impact of vagal hyperactivity. After excluded peptic ulcer patients with HP infection, we found that there was an increased subsequent risk of BPH in non-HP infected peptic ulcer patients either in overall or stratification by sex, age, comorbidity and follow-up intervals (Table 2). Indicating that vagal hyperactivity might play a role in the development of subsequent BPH.

The vagus nerve is the longest autonomic nervous system in the human body and has a larger distribution than any other cranial nerve, which include functions of sensory, motor and parasympathetic, one major function of vagus nerve is its afferent role that brings information of the inner organs (e.g.: gut, liver, heart, and lungs) to the brain, as well as bridge and communication between the brain and inner organs^28,29^. The vagus nerve also play an important role in immunity^30^ and the inflammatory reflex^31,32^. There are epidemiological evidences showed peptic ulcer patients are associated with increased incidences of inflammatory diseases^9–13^. In addition, study also showed over-activity of autonomic nervous system in men with LUTS secondary to BPH^33^, which may indicate the importance of autonomic nervous system in patients with BPH.

The prostate gland is innervated by inferior hypogastric plexus, while the parasympathetic fibers of inferior hypogastric plexus supplies smooth muscle of the pelvic viscera, including prostate^34^. Basically, there were no direct evidences showing that vagus nerve innervate the prostate. However, the pelvic autonomic nervous system is more complex than previously thought. Alsaid et al. demonstrated coexistence of adrenergic and cholinergic nerves in the inferior hypogastric plexus, and the cholinergic fibers could be originated from several sources, including vagus nerves that transmitted via the celiac, mesenteric and superior hypogastric plexuses^35^. Thus, stimulation of cholinergic fibers could result in prostate smooth muscle contraction, which may provide anatomical evidences of the association of persistent vagal hyperactivity and the development of BPH.

On the other hand, steroids such as androgen, testosterone and estrogen may play an important role in immunity^36,37^, and the development of BPH^38^. In addition, studies showed that male sex hormones promote vagally mediated reflex airway responsiveness to cholinergic stimulatio^39^, and gastric vagal afferent is the major pathway conveying ghrelin's signals for starvation and GH secretion to the brain^40^. These results indicate that vagus nerve might play an important role in neuroendocrine reactions^41^. Because vagal system is associated with systemic modulation and homeostasis, although there were few direct evidences delineating the effect of vagus nerve on androgen or androgen receptor, we assumed that the vagus nerve might play a role in a specific neuroendocrine process and androgenic stimulation, which might result in prostate gland overgrowth and the development of BPH. However, further studies are required.

Compared to males without peptic ulcer history, there was a higher incidence and risk of BPH in peptic ulcer patients (Table 2). Additionally, after age-stratified analysis, the risk of BPH was decreased when age increased. The elevated risk of subsequent BPH in peptic ulcer patients with younger age (18–44 years old) may be attributed to the impact of vagal hyperactivity (Table 2).

As we known, the complications of exacerbated peptic ulcer include bleeding, obstruction and perforation, and there is a recent trend for surgical option of peptic ulcer to shift from conventional vagotomy/drainage to simple suture^42^. In the current series, after excluded peptic ulcer patients with HP infection, peptic ulcer patients who underwent simple suture surgery (i.e.: with integrated vagus nerve) showed a significant higher BPH risk than comparison (HR 1.49 and 95% CI 1.28–1.74; SHR 1.26 and 95% CI 1.07–1.48. Table 3). In addition, when stratified by surgery types, patients received simple suture surgery showed a higher incidence and risk of subsequent BPH than patients who underwent TVP (i.e.: with severed vagus nerve) (Table 3). Together, these results may provide further evidences for the association of vagal hyperactivity and BPH.

To the best of our knowledge, there have been no similar studies discussing this issue. Although the mechanisms regarding peptic ulcer and subsequent risk of BPH might be partly speculative, the clinical significance of this study may, at least in part, provide epidemiological evidences of an increased risk of subsequent BPH in peptic ulcer patients, as well as association between gastrointestinal and urological diseases, especially in the Asian population. Furthermore, our finding may spur exploration of the role of the vagus nerve in BPH, and provide suggestions for measurement and possible modulation of vagal activity in male peptic ulcer patients. Since patients with HP infection were excluded, the outcome analysis was limited to non-HP-infected patients and cannot be extrapolated to patients with HP infection.

## Limitation of the study

This long term cohort study is strengthened by the large population with available data regarding longitudinal assessments, as well as subgroup analyses.

Yet, certain limitations are noted. First, because the NIHRD database is an administrative database, variables including lifestyles factors (e.g.: smoking, diet habits, drinking, socioeconomic status, and genetic factors) were not available for adjusting the risk of BPH development. Second, the diagnoses of BPH were based on admission diagnoses and ICD-9 codes, and lack of information in severity/grades or a very precise analysis, as well as lack of biomarkers such as cortisol and inflammation markers IL6, TNFa and IL-1. Third, lack of vagal function test and investigation such as insulin test of gastric secretion, cardiac autonomic function, and neurometry. Fourth, because all data used were anonymous, relevant clinical variables, such as histological findings, imaging results, laboratory data, and measurement of vagal activity (e.g.: heart rate variability) were not available. Fourth, biases from retrospective studies should also be noted.

## Conclusion

In this long-term cohort study, non-HP-infected male peptic ulcer patients were found to have an increased risk of subsequent BPH, which might indicate an important role of vagal hyperactivity. Based on the limitations of retrospective nature, further studies are required.
